# A study on the mechanism of Beclin-1 m6A modification mediated by catalpol in protection against neuronal injury and autophagy following cerebral ischemia

**DOI:** 10.1186/s10020-024-00818-7

**Published:** 2024-05-21

**Authors:** Kan Liu, Xinyan Yao, Jun Gao, Jinxi Wang, Jing Qi

**Affiliations:** 1 Department of Neurology, The First Affiliated Hospital of Hunan University of Chinese Medicine, No. 95 Shaoshan Middle Road, Yuhua District, Changsha, 410007 Hunan People’s Republic of China; 2 Department of Neurosurgery, The First Affiliated Hospital of Hunan University of Chinese Medicine, Changsha, 410007 Hunan People’s Republic of China; 3 Center for Medical Research and Innovation, The First Affiliated Hospital of Hunan University of Chinese Medicine, Changsha, 410007 Hunan People’s Republic of China

**Keywords:** Cerebral ischemia, Catalpol, NRF1, KAT2A, METTL3, Beclin-1, Neuronal injury, Autophagy

## Abstract

**Objective:**

Catalpol (CAT) has various pharmacological activities and plays a protective role in cerebral ischemia. It has been reported that CAT played a protective role in cerebral ischemia by upregulaing NRF1 expression. Bioinformatics analysis reveals that NRF1 can be used as a transcription factor to bind to the histone acetyltransferase KAT2A. However, the role of KAT2A in cerebral ischemia remains to be studied. Therefore, we aimed to investigate the role of CAT in cerebral ischemia and its related mechanism.

**Methods:**

In vitro, a cell model of oxygen and glucose deprivation/reperfusion (OGD/R) was constructed, followed by evaluation of neuronal injury and the expression of METTL3, Beclin-1, NRF1, and KAT2A. In vivo, a MCAO rat model was prepared by means of focal cerebral ischemia, followed by assessment of neurological deficit and brain injury in MCAO rats. Neuronal autophagy was evaluated by observation of autophagosomes in neurons or brain tissues by TEM and detection of the expression of LC3 and p62.

**Results:**

In vivo, CAT reduced the neurological function deficit and infarct volume, inhibited neuronal apoptosis in the cerebral cortex, and significantly improved neuronal injury and excessive autophagy in MCAO rats. In vitro, CAT restored OGD/R-inhibited cell viability, inhibited cell apoptosis, LDH release, and neuronal autophagy. Mechanistically, CAT upregulated NRF1, NRF1 activated METTL3 via KAT2A transcription, and METTL3 inhibited Beclin-1 via m^6^A modification.

**Conclusion:**

CAT activated the NRF1/KAT2A/METTL3 axis and downregulated Beclin-1 expression, thus relieving neuronal injury and excessive autophagy after cerebral ischemia.

## Introduction

Broadly speaking, cerebral ischemia refers to a syndrome in which the brain blood supply is insufficient and it is difficult to meet the metabolic needs of brain tissue, resulting in a series of symptoms (Ahad et al. 2020). Severe cerebral ischemia, mostly in the elderly, can cause irreversible damage to brain function and even death (Kaviarasi et al. 2019). Recently, many efforts have been made in the treatment of cerebral ischemia, mainly in the direction of natural medicines and pathophysiological signaling pathways, but the prospect is not satisfactory (Qin et al. 2022; Tao et al. 2020). Intrinsically, the survival of neurons greatly affects the integrity and stability of brain function, and their loss can directly lead to cerebral functional deficits, indicating the indispensable role of neurons in the treatment of cerebral ischemia (Zhao et al. 2022). Moreover, researchers found that neuronal autophagy is activated upon ischemic stroke and is widely involved in the progression of cerebral ischemia (Zhang et al. 2020). Therefore, it is desirable to study the related mechanism in the direction of neuronal protection.

Catalpol (CAT), isolated from *Rehmannia glutinosa*, has many pharmacological activities including anti-oxidative and anti-inflammatory effects and exerts a protective role in cerebral ischemia (Wang et al. 2022). A study on the mechanism of CAT in cerebral ischemia stated that CAT protected vascular structure and promoted angiogenesis in focal cerebral ischemic rats by activating the hypoxia-inducible factor 1-alpha (HIF-1α)/vascular endothelial growth factor (VEGF) pathway (Wang et al. 2020). It was noted that CAT prevented muscle atrophy through the regulation of muscle apoptosis and autophagy via the mTOR pathway (Wang et al. 2019a, b). CAT may promote axon growth by regulating the phosphoinositide 3-kinase/v-akt murine thymoma viral oncogene homolog/mechanistic target of rapamycin pathway regulated by miR-124 after neuronal ischemia (Zhu et al. 2019a, b). Importantly, CAT increased the length of microtubule-associated protein 2 (MAP-2) positive neurites and the expression of synaptic proteins, and promoted neuron recovery, assuming protective roles in Aβ-induced neuronal injury and cognitive dysfunctions in aged rats (Xia et al. 2017). Nevertheless, the potential effect of CAT on neuronal autophagy in cerebral ischemia is still not completely clear.

It has been documented that CAT can increase the expression of nuclear respiratory factor 1 (NRF1) (Chen et al. 2022), and elevated NRF1 can improve cognitive performance and play a protective role in rat models of ischemic encephalopathy (Wang et al. 2023). According to the prediction of hTFtarget database, NRF1 can bind to lysine acetyltransferase 2A (KAT2A). As a well-known histone acetyltransferase, KAT2A has been reported to have non-histone acetyltransferase activity and contribute to axon growth and neurogenesis (Lin et al. 2022). A related study revealed that KAT2A played a protective role in myocardial ischemia–reperfusion (Lei et al. 2021). Hence, we speculated that NRF1 may protect against ischemic injury by promoting KAT2A transcription.

Moreover, KAT2A could increase the H3K27 acetylation level in the METTL3 promoter region to activate METTL3 transcription (Liao et al. 2022). Increasing data present that METTL3 is an m^6^A transferase that plays a protective role in cerebral ischemia (Liu et al. 2023; Si et al. 2020). SRAMP database showed multiple m^6^A modification sites on Beclin-1 mRNA, and RM2Target database predicted that METTL3 could bind to Beclin-1 mRNA. Reportedly, loss of Beclin-1 could inhibit autophagy activation and prevent secondary neurodegenerative damage to the ipsilateral thalamus after focal cerebral infarction (Xing et al. 2012). On this basis, we guessed that METTL3 may affect Beclin-1 mRNA expression by regulating the m^6^A level. Hence, we speculated that CAT may promote METTL3 transcription by up-regulating NRF1/KAT2A, regulates m^6^A level of Beclin-1, and protect against cerebral ischemic process. Accordingly, we analyzed the mechanism of CAT in cerebral ischemic via the NRF1/KAT2A/METTL3/Beclin-1 axis with the focus on neuronal injury and autophagy, aiming to offer a theoretical basis for the development of CAT as neuroprotective drugs for the prevention and treatment of cerebral ischemia.

## Materials and methods

### Establishment of middle cerebral artery occlusion (MCAO) model and grouping

Male Sprague–Dawley rats obtained from Vital River Laboratory Animal Technology Co., Ltd. (Beijing, China) were raised under conditions of 20–25 °C temperature and 60–70% humidity with a 12-h light/dark cycle at least 1 week and free access to food and water. All animal experiments in this study were approved by the Ethics Committee of our hospital [No. (ZYFY20200820-07)], and the experimental procedures were executed in accordance with the National Institutes of Health Experimental Animal Care and Use Guidelines.

Rats were allocated into 5 groups as follows: (1) sham group (n = 12): rats underwent the same operation as the experimental rats, nylon monofilament insertion, and no treatment with CAT or normal saline; (2) MCAO group (n = 12): MCAO rat model was prepared by means of focal cerebral ischemia as previously described (Pengyue et al. 2017). In short, after anesthesia, the left common carotid artery (CCA), external carotid artery (ECA), and internal carotid artery (ICA) of rats were separated from adjacent muscles and nerves, respectively. Nylon monofilament was inserted through a small incision in the ECA, backed into the CCA, and then gently inserted into the middle cerebral artery (MCA) from the ICA. After MCAO for 90 min, nylon monofilament was removed anf reperfusion was performed; (3) MCAO + CAT group (n = 24): based on the study by Zhu et al. (2019) and Dong et al. (2016), 6 h after ischemia, rats received daily intraperitoneal administration with CAT (5 mg/kg) for 7 days; (4) MCAO + CAT + LV-NC group (n = 12): rats were injected with the negative control of interfering Lentiviral vector of NRF1 (LV-NRF1) (LV-NC) and then underwent MCAO and CAT treatments; (5) MCAO + CAT + LV-NRF1 group (n = 12): rats were injected with LV-NRF1 and then underwent MCAO and CAT treatments. CAT (purity ≥ 96%) was purchased from Sigma Aldrich (Shanghai, China), and its reserve solution was prepared in saline.

### Lentivirus injection

The location of the cerebral motor cortex (3.7 mm anterior to the bregma, 2.0 mm lateral to the midline, 1.0 mm below the dural surface) was determined by reference to the stereotaxic atlas of the rat brain: the Rat Brain in Stereotaxic Coordinates-3th Edition. Interfering lentiviral vector of NRF1 and its negative control (LV-NRF1 and LV-NC) were constructed by Hanbio Biotechnology (Shanghai, China), which were injected into rat brain according to the groups by using a microsampler. The injection was completed within 2 min, and the needle was left for 30 s. Following 3 consecutive days of injection, MCAO rat model was prepared on the 4th day.

### Neurological deficit score

In order to evaluate the effect of CAT on ischemia-caused neurological deficits, the neurological deficit was scored after 7 days of MCAO treatment with the following criteria (Yang et al. 2021a, b): 0 scores, no obvious neurological deficit; 1 score, slight forelimb weakness; 2 scores, moderate forelimb weakness, and turning to the deficit side when tail lifting; 3 scores, severe neurological deficit, and turning to the hemiplegia side while walking; 4 scores, difficulty or unable to walk autonomously.

### Determinations of cerebral infarct volume and brain water content (BWC)

After neurological deficit scoring, rats were anesthetized via intraperitoneal injection with sodium pentobarbital (50 mg/kg) and then euthanized by cervical devertebrae. Afterward, their brains were removed, followed by frozen at − 20 °C and the preparation of 2 mm thick sections. Next, the sections were stained in 2% triphenyl tetrazolium chloride (TTC) solution (Genink Biotechnology, Tianjin, China) at 37 °C for 30 min and then fixed in 4% paraformaldehyde at room temperature for 12 h. Following TTC staining, infracted brain tissue was light color, while unstained brain tissue was red. The infarct size was measured by ImageJ analysis software, and then the percentages of infarct volume and BWC were calculated as the following formulas: 

${\text{Infarct volume }}\left( \% \right)\, = \,{\text{infarct volume}}/{\text{total brain volume}}\, \times \,{1}00\%$ (Deng et al. 2021);

${\text{BWC }}\left( \% \right)\, = \,\left( {{\text{wet weight}}\, - \,{\text{dry weight}}} \right)/{\text{wet weight}}\, \times \,{1}00\%$ (Wei et al. 2021).

### Histopathological evaluation

After neurological deficit scoring, rat brain was infused with 4% precooled paraformaldehyde in 0.1 M phosphate-buffered saline (PBS), followed by the removal of the brain for soaking in paraformaldehyde overnight. The brain mass containing the prefrontal cortex region was embedded with paraffin wax and then cut into 6-μm coronal sections using a microtome. The sections underwent hematoxylin and eosin (H&E) and Nissl staining, followed by observation under a light microscope (Olympus, Tokyo, Japan).

### TUNEL assay for tissue apoptosis

This assay was performed according to the instructions of cell death test kits (Roche Molecular Biochemicals, Mannheim, Germany). The paraffin-embedded sections were reacted with protease-K (37 °C, 15 min), followed by PBS washing and incubation in 0.3% hydrogen peroxide. Thereby, the sections were incubated with TUNEL reaction mixture (a mixture of 50 μL TdT + 450 μL fluorescein-labeled dUTP) at 37 °C for 1 h in dark. Finally, the apoptotic cells were observed and counted under a fluorescence microscope (Olympus).

### Transmission electron microscopy (TEM)

The cortical brain tissue or cells of each group were fixed with 4% osmium tetroxide in 0.1 M PBS, dehydrated in graded ethanol, cleared in propylene oxide, and permeated with an epoxy-plastic mixture. After that, the tissues or cells were saturated with epoxy resin and sliced. The sections underwent double staining with lead citrate and uranyl acetate. Images of the sections were obtained through TEM (JEM-2200FS TEM26, Tokyo, Iapan), and the number of autophagosomes in the neurons was counted.

### Immunofluorescent staining

The coexpression of NeuN (neuronal marker) and LC3 (autophagy marker) antibodies in ischemic brain tissue of rats was analyzed through immunofluorescent staining. In short, the sections of brain tissues were treated with NeuN murine monoclonal antibody (66836-1-Ig, 1:50, Proteintech, Chicago, IL, USA) and LC3 rabbit polyclonal antibody (#4108, 1:1000, Cell Signaling Technology, Boston, MA, USA) at 4 °C overnight. After PBS rising, the sections were incubated with fluorescent secondary antibody at 37 °C for 1 h. The nuclei were stained with DAPI, and the images were captured using a fluorescence microscope.

Cells were fixed in 4% paraformaldehyde for 10 min and washed in PBS, followed by overnight incubation (4 °C) with rabbit anti-LC3 antibody and then 1-h incubation (37 °C) with fluorescent secondary antibody. After DAPI staining, a fluorescence microscope was used to collect images.

### Cell culture and transfection

The rat cortical neurons (RN-c, MZ-7885) were purchased from Mingzhou Biotechnology (Ningbo, China), and the RN-c cell complete culture medium was from ORiCells Biotechnology (Shanghai, China).

Cell transfection was conducted based on the instructions of riboFECTTMCP transfection kit produced by RiboBio (Guangzhou, China), followed by 6-h incubation before subsequent experiments. Beclin-1 overexpression and interference plasmids (oe-Beclin-1 and sh-Beclin-1), METTL3 overexpression and interference plasmids (oe-METTL3 and sh-METTL3), KAT2A overexpression and interference plasmids (oe-KAT2A and sh-KAT2A), NRF1 interference plasmid (sh-NRF1), and their negative controls (oe-NCs and sh-NCs) were obtained from GenePharma (Shanghai, China).

### A cell model of oxygen and glucose deprivation/reperfusion (OGD/R) and CAT treatment

As previously described (Sun et al. 2018), OGD/R was executed to simulate cerebral ischemia reperfusion injury in vitro. RN-c cells were washed twice with PBS (pH = 7.4) and then refreshed in glucose-free DMEM (Gibco, Grand Island, USA). Afterward, cells were transferred to a hypoxia chamber (a gas mixture including 5% CO_2_ and 95% N_2_) at 37 °C. After 4 h of incubation, the RN-c cells underwent incubation in Neurobasic medium containing 0.5 mM glutamine and 2% B27 and then returned to a normoxic incubator (95% air and 5% CO_2_) for 24 h at 37 °C. The untreated cells served as control group (CON group). For CAT treatment, 10 μg/mL of CAT was added to the medium during OGD and the culture was maintained until the end of OGD/R (Wang et al. 2019a, b; Zhu et al. 2019a, b).

### Cell counting kit 8 (CCK-8) for cell viability

After RN-c cells were incubated with CCK-8 kit (Beyotime, Shanghai, China) for 4 h, the absorbance was measured at the wavelength of 450 nm on a microplate reader to test cell viability.

### Lactic dehydrogenase (LDH) measurement

Lactate dehydrogenase (LDH) is released from RN-c cells when they are damaged, so the amount of LDH in the medium is an indicator of membrane integrity. The LDH activity in cells was quantified by a LDH cytotoxicity assay kit (Sigma-Aldrich). The absorbance was examined at the wavelength of 490 nm on a microplate reader. Finally, the amount of LDH released was expressed as a percentage of total LDH to assess RN-c cell injury exposed to OGD/R.

### Flow cytometry for cell apoptosis

Cells were washed with pre-cooled PBS and then resuspended in Annexin V binding buffer. Next, cell suspension was stained with Annexin V-fluorescein isothiocyanate (FITC)/propidium iodide (PI) and placed in dark at room temperature for 15 min, which was transferred onto ice. The apoptosis rate was tested by flow cytometry within 1 h according to the instructions of Annexin V-FITC/PI kits (TransGen Biotech, Beijing, China).

### Western blotting

Referring to the manufacturer's instructions, the protein was extracted from tissues or cells using a total protein extraction kit (Solarbio, Beijing, China). Protein quantification was performed with a bicinchoninic acid kit (Solarbio). Subsequent to electrophoresis, the protein was transferred onto a polyvinylidene fluoride (PVDF) membrane. After sealing in 5% skimmed milk, the membrane was probed with primary antibodies anti-NRF1 (#46743, Cell Signaling Technology), anti-KAT2A (66575-1-Ig, Proteintech), anti-METTL3 (ab195352, Abcam, Cambridge, UK), anti-LC3 (#4108, Cell Signaling Technology), anti-p62 (ab211324, Abcam), anti-Beclin-1 (ab302669, Abcam), and anti-β-actin (GTX109639, Genetex, Irvine, CA, USA) at 4 °C overnight. Afterward, the membrane was re-probed with secondary antibody for 2 h and washed with PBS (3 × 10 min). Following addition of developer solution, the membrane was examined using a chemiluminescent imaging system.

### Reverse transcription-quantitative polymerase chain reaction (RT-qPCR)

The total RNA was isolated using TRIzol (Thermo Fisher Scientific, Waltham, MA, USA). RT-qPCR was performed on the Applied Biosystems 7500 real-time PCR system using SuperScript™ III Platinum™ SYBR™ Green one-step kits (Thermo Fisher Scientific). The relative expression of target genes was calculated with β-actin as the internal reference using the 2^−ΔΔCt^ method. Primers are shown in Table 1.

**Table 1:** Primer sequences used in reverse transcription-quantitative polymerase chain reaction analysis

Name of primer	Sequences (5′-3′)
β-actin-F	CCGTAAAGACCTCTATGCCAACA
β-actin-R	CGGACTCATCGTACTCCTGCT
NRF1-F	TACAAGGCGGGGGACAGATA
NRF1-R	TGCATGAACTCCATCTGGGC
KAT2A-F	GTTCCTGTCCATGCTTGAGGA
KAT2A-R	AGCTTCCTCTTCTCTCCTGGCA
METTL3-F	ATCCCCAAGGCTTCAACCAG
METTL3-R	ATCCAGTTGGGCTGCACATT
Beclin-1-F	GAATGGAGGGGTCTAAGGCG
Beclin-1-R	CTTCCTCCTGGCTCTCTCCT

*F* forward, *R* reverse

### Dual-luciferase reporter gene assay

The binding sites between NRF1 and KAT2A promoter were predicted through online prediction software JASPAR (https://jaspar.genereg.net/). The wild and mutant sequences of KAT2A containing NRF1 binding sites (mut-KAT2A and wt-KAT2A) were designed and synthesized based on the prediction results, which were inserted into pGL3-Basic vectors (Promega, Wisconsin, USA) and co-transfected with oe-NRF1 or oe-NC into cells. After 48 h, the fluorescence activity intensity was detected using luciferase reporter gene kits (YEASEN, Shanghai, China).

### Chromatin immunoprecipitation (ChIP)

ChIP assay was conducted using EZ ChIP kits (Millipore, Darmstadt, Germany) on the basis of the instructions. Cells were subjected to 10-min crosslinking with 1% formaldehyde, which was terminated using glycine. Next, the cells were lysed in sodium dodecyl sulfate lysis solution, and the obtained lysate underwent DNA cutting using an ultrasonic crusher, with the fragment length between 200 and 1000 bp. Anti-NRF1 (sc-28310, Santa Cruz Biotechnology, CA, USA) and H3K27AC (ab4729, Abcam) were employed for ChIP assay. Finally, the purified DNA products were examined by qPCR.

### Methylated RNA IP (MeRIP)

This experiment was performed using Magna MeRIP m^6^A kits (Millipore). Specifically, the anti-m^6^A antibody (ab208577, Abcam) or anti-IgG antibody was supplemented into IP buffer for 1 h for binding to protein A/G magnetic beads. The purified mRNA and magnetic bead-antibody complex were mixed with IP buffer containing ribonucnase inhibitors and protease inhibitors, followed by overnight incubation at 4 °C. After the eluted RNA was purified by phenol–chloroform, the IP products were utilized for qPCR.

### Photoactivatable ribonucleoside-enhanced crosslinking and IP (PAR-CLIP)

RN-c cells underwent 14-h incubation with 200 mm 4-thiopyridine (Sigma Aldrich) and then crosslinking at 365 nm with 0.4 J/cm^2^. Following lysis, anti-METTL3 (ab195352, Abcam) was used for IP at 4 °C. The precipitated RNA was labeled with [g-32-P]-ATP and observed through autoradiography. After digestion with protease K for protein removal, RNA was extracted for measurement of Beclin-1 mRNA expression by qPCR.

### RNA stability analysis

Normal neurons were transfected with sh-NC or sh-METTL3 and then treated with actinomycin D (final concentration: 1 µg/mL) to stop mRNA transcription. Cells were gathered at specified time points (0, 3, 6, and 9 h) to assess mRNA degradation, followed by RNA extraction for the analysis of the relative mRNA level of Beclin-1 by qPCR.

### Statistical analysis

All statistical analyses were conducted using GraphPad Prism8.02 software. Data from at least three independent assays were presented as mean ± standard deviation. Normality and variance homogeneity test were conducted: Shapiro–Wilk test was used to analyze the legitimacy of the data, and Bartlett test the variance homogeneity of the analyzed data. Unpaired *t*-test was used to compare data between the two groups, and one-way or two-way ANOVA was employed for comparisons among multiple groups, with the Tukey's multiple comparisons test used for post hoc analysis. *, #, or & meant *P* < 0.05, which was thought to have statistical significance.

## Results

### CAT improves neuronal injury and excessive autophagy in MCAO rats

**Fig. 1:**
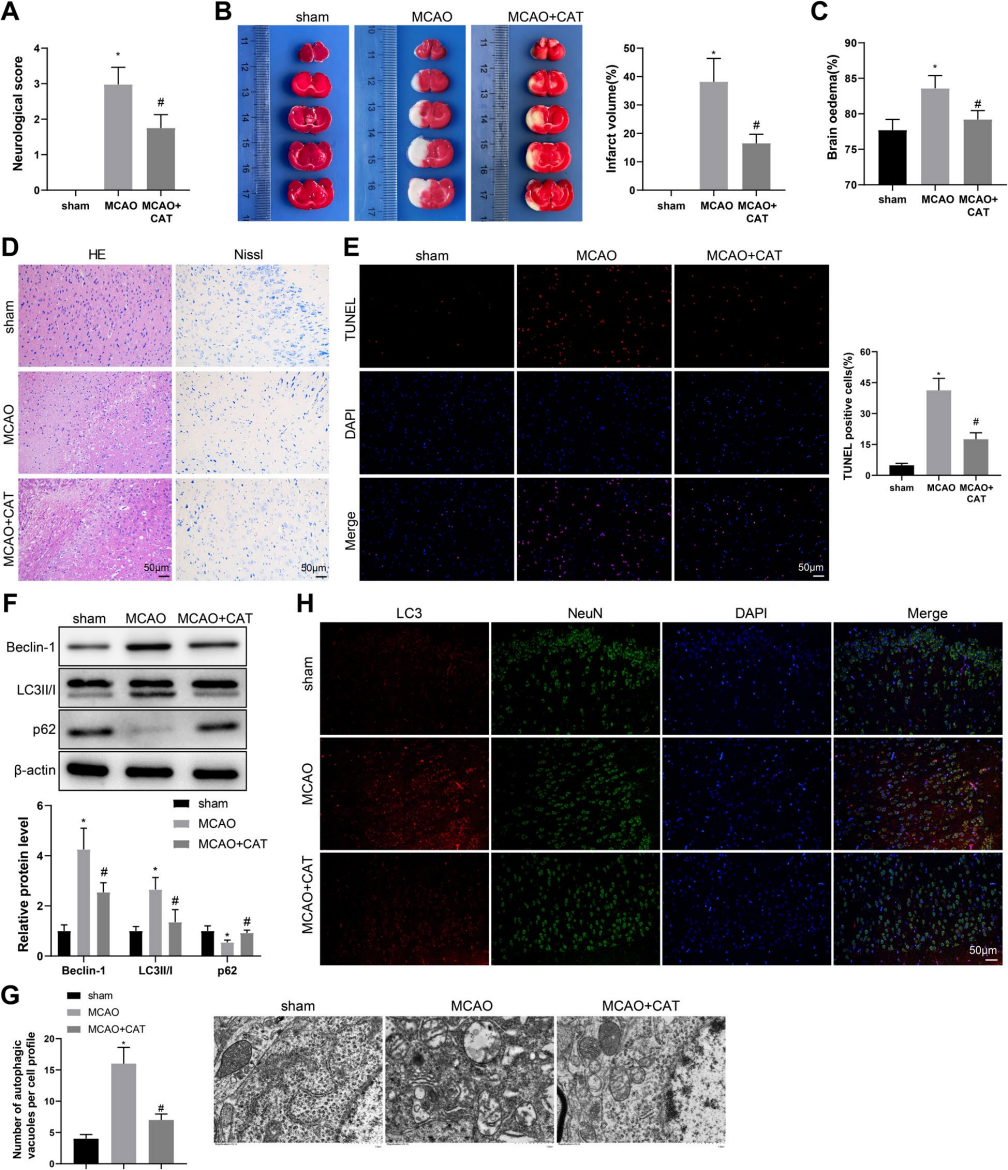
CAT treatment relieves neuronal injury and excessive autophagy in MCAO rats. **A** Neurological deficit score (n = 12). **B** The infarct volume was measured through TTC staining (n = 6). **C** BWC was examined (n = 6). **D** H&E staining and Nissl staining were used to evaluate the morphological changes in the prefrontal cortex (n = 6). **E** TUNEL staining was used to determine the apoptosis of cortical neurons (n = 6). **F** The protein expression of Beclin-1, LC3II/I, and p62 in ischemic cerebral cortex was tested by western blotting (n = 6). **G** The number of autophagosomes in cortical neurons was observed through TEM (n = 6). **H** The representative images of LC3, NeuN, and DAPI (blue) immunofluorescent staining of cortical areas in each group of rats (n = 6). *P < 0.05, compared with the sham group; #*P* < 0.05, compared with the MCAO group

First, we established a rat model of MCAO and injected CAT for intervention to explore whether CAT can regulate cerebral cortex autophagy and improve neuronal damage after cerebral ischemia. Neurological deficit score showed that rats in the MCAO group had increased scores versus the sham group, while CAT treatment greatly declined the scores in MCAO rats, indicating better neurobehavioral outcomes (Fig. 1A). As reflected in Fig. 1B, C, the infarct volume and BWC were higher in the MCAO group than those in the sham group, which were reduced by CAT treatment. Results of H&E staining revealed that the rats in the sham group had normal tissues with tightly ordered prefrontal cortexes and visible nuclei, while most disordered cells and dense or severely atrophied nuclei were observed in MCAO rats (Fig. 1D). Nissl staining displayed that neurons in the sham group were intact and the cell body was full, but the proportion of Nissl-positive cells in the MCAO group was reduced, accompanied by atrophied neurons and shrunken nuclei (Fig. 1D). Expectedly, CAT treatment had a protective effect against neuronal injury, which was manifested as less cell injury, clearer nuclei, more Nissl-positive cells, and less atrophied neurons in the MCAO + CAT group (Fig. 1D). Additionally, data from TUNEL staining exhibited that the MCAO group had more TUNEL-positive cells than the sham group, which was reversed by CAT treatment (Fig. 1E).

Western blotting unraveled that the MCAO group had elevated protein levels of Beclin-1 and LC3II/I and reduced protein level of p62 versus the sham group, which were reversed by CAT treatment (Fig. 1F). Through TEM, it was observed that the number of autophagosomes in cortical neurons was significantly increased in the MCAO group versus the sham group but decreased in the MCAO + CAT group versus the MCAO group (Fig. 1G), which suggested that CAT treatment could inhibit excessive neuronal autophagy in MCAO rats, consistent with immunofluorescent staining results (Fig. 1H). Above results demonstrated that CAT treatment could significantly mitigate neuronal injury and inhibit excessive autophagy in MCAO rats after ischemia.

### CAT alleviates OGD/R-induced neuronal injury and excessive autophagy by inhibiting Beclin-1

**Fig. 2:**
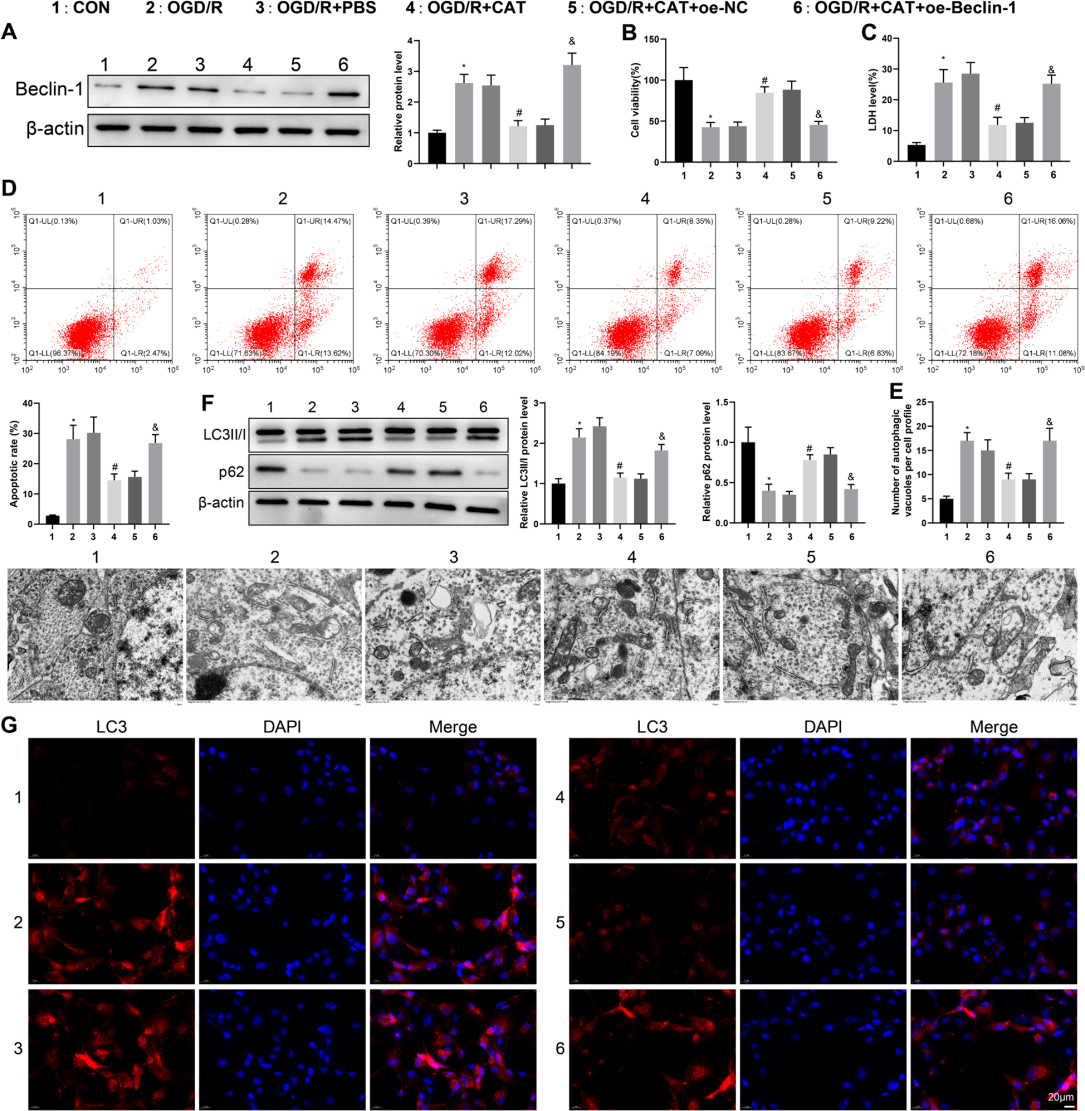
CAT exerts protective effects against OGD/R-induced neuronal injury and excessive autophagy via Beclin-1. **A** The expression of Beclin-1 was measured by western blotting. **B** RN-c cell viability was tested by CCK-8 assay. **C** LDH release was evaluated using the kits. **D** RN-c cell apoptosis was detected by flow cytometry. **E** The number of autophagosomes in RN-c cells was observed through TEM. **F** The expression of LC3II/I and p62 was assessed by western blotting. **G** The representative images of immunofluorescent staining of LC3. Cell experiments were repeated three times. *P < 0.05, compared with the CON group; #*P* < 0.05, compared with the OGD/R + PBS group; &*P* < 0.05, compared with the OGD/R + CAT + oe-NC group

As reported, Beclin-1 was linked to OGD/R-triggered autophagic cell death (Fan et al. 2016). Combining all the above findings, we speculated that CAT may regulate autophagy and play a neuroprotective role in cerebral ischemia through Beclin-1. To verify this speculation, after oe-Beclin-1 or oe-NC transfection, RN-c cells were treated with OGD/R to simulate cerebral ischemia in vitro, during which the cells underwent CAT treatment. Compared with the CON group, the protein expression of Beclin-1 in RN-c cells was increased in the OGD/R group; compared with the OGD/R + PBS group, Beclin-1 expression was decreased in the OGD/R + CAT group; however, oe-Beclin-1 transfection augmented Beclin-1 expression in the OGD/R + CAT + oe-Beclin-1 group versus the OGD/R + CAT + oe-NC group (Fig. 2A).

First, we evaluated the effects of CAT and Beclin-1 on cortical neuronal injury by detecting cell viability, apoptosis, and LDH release. CCK-8 assay elicited that RN-c cell viability was weakened in the OGD/R group (vs. the CON group) but enhanced in the OGD/R + CAT group (vs. the OGD/R + PBS group), while the OGD/R + CAT + oe-Beclin-1 group had lower RN-c cell viability than the OGD/R + CAT + oe-NC group (Fig. 2B). LDH activity kits and flow cytometry clarified that RN-c cell apoptosis and LDH activity were higher in the OGD/R group than that in the CON group but lower in the OGD/R + CAT group than that in the OGD/R + PBS group, while RN-c cell apoptosis and LDH activity were elevated by oe-Beclin-1 transcription (Fig. 2C, D). These results suggested that CAT exerted neuroprotective effects after cerebral ischemia through Beclin-1.

Subsequently, we evaluated the autophagy regulation of CAT and Beclin-1 in RN-c cells. Observations from TEM showed that the OGD/R group had more autophagosomes than the CON group, the OGD/R + CAT group had less autophagosomes than the OGD/R + PBS group, and the OGD/R + CAT + oe-Beclin-1 group had more autophagosomes than the OGD/R + CAT + oe-NC group (Fig. 2E). Western blotting revealed that the OGD/R group had increased LC3II/I and decreased p62 compared with the CON group; the OGD/R + CAT group had decreased LC3II/I and increased p62 versus the OGD/R + PBS group; however, the OGD/R + CAT + oe-Beclin-1 group had elevated LC3II/I and reduced p62 related to the OGD/R + CAT + oe-NC group (Fig. 2F). Analysis of immunofluorescent staining reflected that the number of LC3 spots was increased in the OGD/R group (vs. the CON group) but decreased in the OGD/R + CAT group (vs. the OGD/R + PBS group), while oe-Beclin-1 transfection boosted the number of LC3 spots (Fig. 2G). Taken together, CAT exerted the protective effect and autophagy regulation of cortical neuronal injury after cerebral ischemia via Beclin-1.

### METTL3 mediates m^6^A modification of Beclin-1

**Fig. 3:**
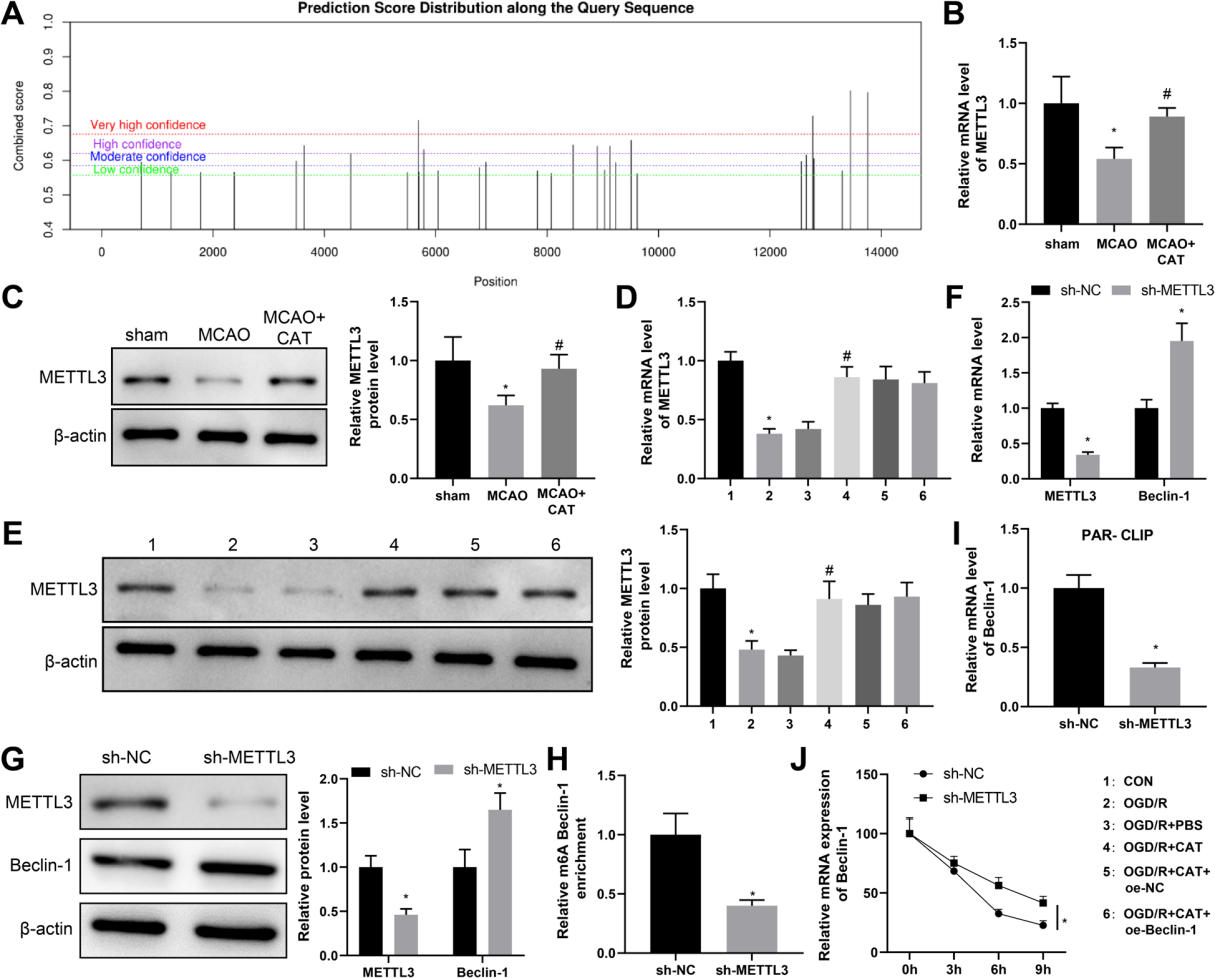
METTL3 negatively regulates Beclin-1 expression by regulating the m^6^A level of Beclin-1 mRNA. **A** SRAMP database was used to predict the m^6^A modification sites on Beclin-1 mRNA. **B**–**E** The expression of METTL3 in the brain tissues of MACO rats and OGD/R-induced RN-c cells was examined by RT-qPCR and western blotting. After sh-METTL3 transfection, **F**, **G** the expression of METTL3 and Beclin-1 was assessed by RT-qPCR and western blotting. **H** the m^6^A modification level of Beclin-1 was examined by Me-RIP assay. **I** the mRNA binding of METTL3 and Beclin-1 was detected by PAR-CLIP assay. **J** the stability of Beclin-1 mRNA was determined by actinomycin D assay. Cell experiments were repeated three times, and animal experiments: n = 6. *P < 0.05, compared with the sham, CON, or sh-NC group; #*P* < 0.05, compared with the MACO or OGD/R + PBS group

Next, SRAMP database showed that there were multiple m^6^A modification sites on Beclin-1 mRNA (Fig. 3A). RM2Target database predicted that METTL3 could bind to Beclin-1 mRNA. As for the findings of RT-qPCR and western blotting, METTL3 was substantially decreased in OGD/R-induced RN-c cells and the brain tissues of MACO rats, which could be enhanced by CAT treatment (Fig. 3B–E). Of note, the change of Beclin-1 expression did not affect METTL3 expression in RN-c cells (Fig. 3D, E). Next, we transfected sh-METTL3 into normal neurons and did RT-qPCR and western blotting assays, which manifested that the sh-METTL3 group had decreased expression of METTL3 and increased expression of Beclin-1 compared with the sh-NC group (Fig. 3F, G). Therefore, we speculated that METTL3 might negatively regulate Beclin-1 expression by regulating the m^6^A level of Beclin-1 mRNA.

Thereby, m^6^A modification level of Beclin-1 was detected by Me-RIP assay, and the results exhibited that the m^6^A modification level of Beclin-1 in RN-c cells of the sh-METTL3 group was prominently reduced compared with that of the sh-NC group (Fig. 3H). Furthermore, PAR-CLIP results discovered that the mRNA expression level of Beclin-1 pulled down by METTL3 antibody was markedly decreased after sh-METTL3 transfection (Fig. 3I). The actinomycin D experiment unveiled that the sh-METTL3 group had higher Beclin-1 mRNA stability than the sh-NC group (Fig. 3J). Above findings demonstrated that METTL3 knockdown facilitated the expression of Beclin-1 by inhibiting the m^6^A modification level of Beclin-1.

### CAT mitigates OGD/R-induced neuronal injury and excessive autophagy via the inhibition of Beclin-1 mediated by METTL3

**Fig. 4:**
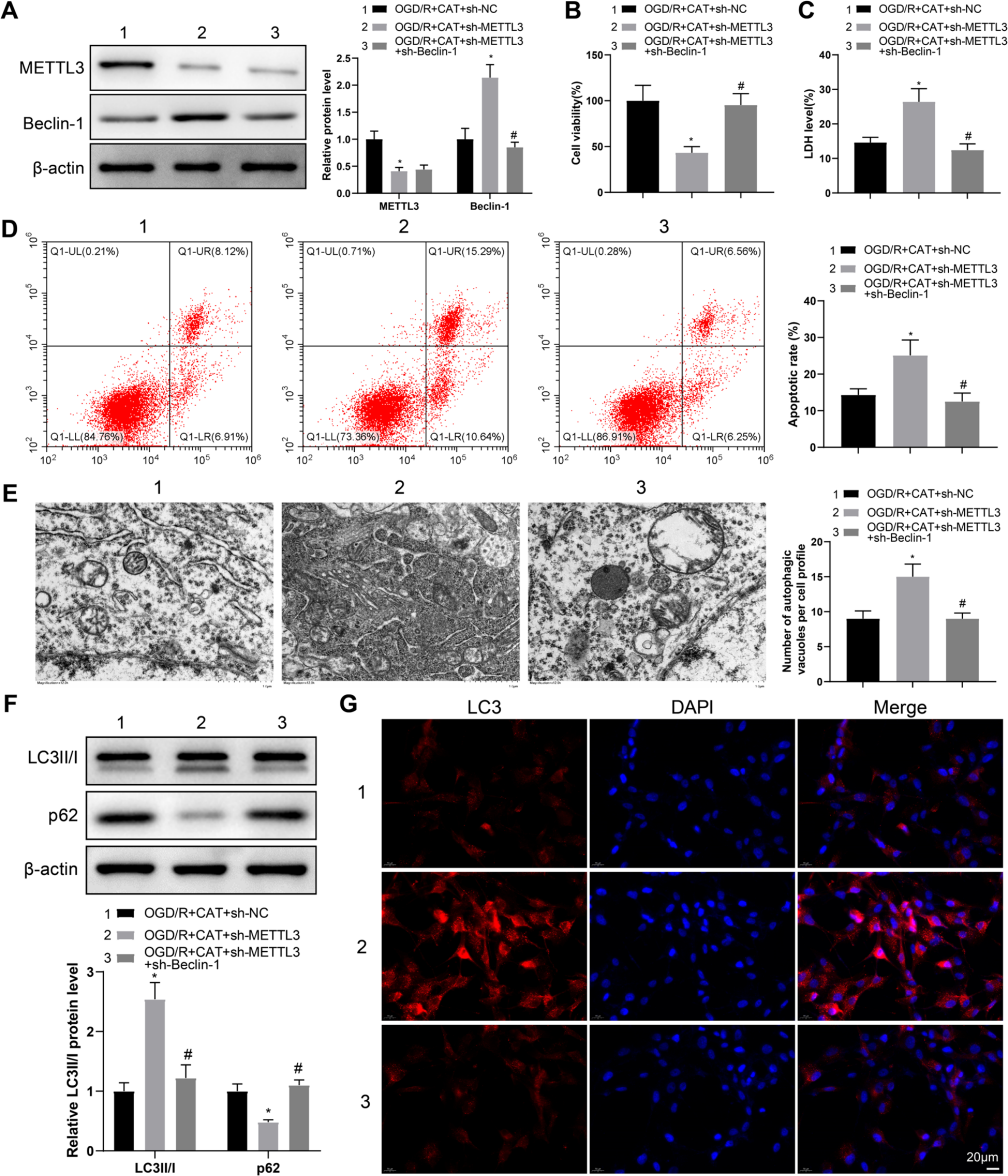
CAT improves OGD/R-triggered neuronal injury and excessive autophagy via METTL3/Beclin-1. **A** the protein expression of METTL3 and Beclin-1 in RN-c cells was measured by western blotting. **B** RN-c cell viability was detected by CCK-8 assay. **C** LDH release was evaluated using the kits. **D** RN-c cell apoptosis was tested by flow cytometry. **E** the number of autophagosomes in RN-c cells was observed through TEM. **F** the expression of LC3II/I and p62 was examined by western blotting. **G** the representative images of immunofluorescent staining of LC3. Cell experiments were repeated three times. *P < 0.05, compared with the OGD/R + CAT + sh-NC group; #*P* < 0.05, compared with the OGD/R + CAT + sh-METTL3 group

To delve into whether CAT assume a role in the process of cerebral ischemia through METTL3 mediated Beclin-1 m^6^A modification, RN-c cells received transfection with sh-METTL3 and sh-Beclin-1 and then OGD/R treatment, accompanied by CAT treatment during OGD/R. In comparison with the OGD/R + CAT + sh-NC group, decreased METTL3 expression and increased Beclin-1 expression were noticed in the OGD/R + CAT + sh-METTL3 group, while the protein expression of Beclin-1 was repressed in the OGD/R + CAT + sh-METTL3 + sh-Beclin-1 group versus the OGD/R + CAT + sh-METTL3 group, with no change in METTL3 expression (Fig. 4A).

With regard to CCK-8 results, the OGD/R + CAT + sh-METTL3 group had lower RN-c cell viability than the OGD/R + CAT + sh-NC group, but the OGD/R + CAT + sh-METTL3 + sh-Beclin-1 group had higher RN-c cell viability than the OGD/R + CAT + sh-METTL3 group (Fig. 4B), which was contrary to the results of LDH release and cell apoptosis (Fig. 4C, D).

In contrast to the OGD/R + CAT + sh-NC group, the number of autophagosomes in RN-c cells was enhanced in the OGD/R + CAT + sh-METTL3 group, which was reduced by further transfection of sh-Beclin-1 (Fig. 4E). As expected, the OGD/R + CAT + sh-METTL3 group had higher LC3II/I expression but lower p62 expression than the OGD/R + CAT + sh-NC group, while the opposite results were noted in the OGD/R + CAT + sh-METTL3 + sh-Beclin-1 group when compared with the OGD/R + CAT + sh-METTL3 group (Fig. 4F). Consistently, the same changes of autophagy were found through LC3 immunofluorescent staining (Fig. 4G). Conclusively, CAT inhibited Beclin-1 expression via m^6^A modification of Beclin-1 mediated by METTL3, thereby improving OGD/R-induced cortical neuronal injury and inhibiting excessive autophagy.

### NRF1 activates METTL3 transcription via KAT2A

**Fig. 5:**
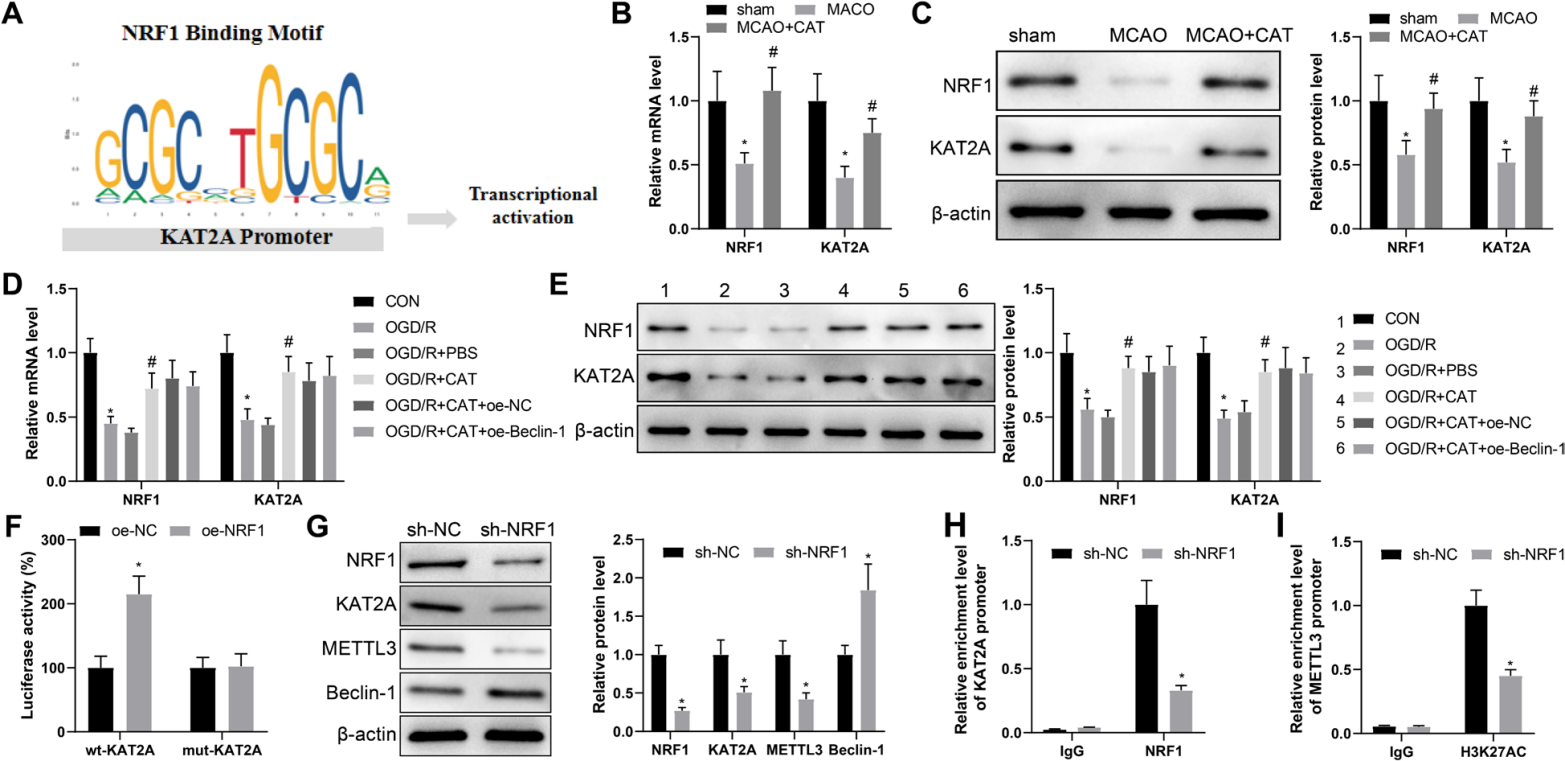
NRF1 enhances METTL3 transcription via KAT2A. **A** The binding relationship between NRF1 and KAT2A was predicted by JASPER database. **B**–**E** the mRNA and protein expression of NRF1 and KAT2A in the brain tissues of MACO rats and OGD/R-induced RN-c cells was assessed by RT-qPCR and western blotting. **F** The combination of NRF1 and KAT2A was tested by dual-luciferase reporter gene assay. After sh-NRF1 transfection in normal neurons, **G** western blotting was used to measure the protein expression of NRF1, KAT2A, METTL3, and Beclin-1. **H** The enrichment level of KAT2A promoter region pulled down by NRF1 antibody was detected by ChIP. **I** the enrichment level of H3K27AC in the METTL3 promoter region was detected by ChIP. Cell experiments were repeated three times, and animal experiments: n = 6. *P < 0.05, compared with the sham, CON, oe-NC, or sh-NC group; #*P* < 0.05, compared with the MACO or OGD/R + PBS group

Liao et al. pointed out that KAT2A could increase the H3K27 acetylation level in the METTL3 promoter region to activate METTL3 transcription (Liao et al. 2022). A prior study reported that KAT2A played a protective role in myocardial ischemia–reperfusion (Lei et al. 2021); however, its role in cerebral ischemia remains to be disclosed. Moreover, CAT has been reported to upregulate NRF1 expression (Chen et al. 2022), and overexpressed NRF1 played a protective role in ischemic encephalopathy (Wang et al. 2023). In this paper, JASPER database predicted that NRF1 could bind to KAT2A as a transcription factor (Fig. 5A). On this basis, we conjectured that NRF1 may activate METTL3 transcription by promoting KAT2A transcription and increasing H3K27 acetylation level in METTL3 promoter region, thus playing a protective role in cerebral ischemia.

To validate our hypothesis, RT-qPCR and western blotting were designed to detect the expression of NRF1 and KAT2A, which illustrated reduced expression of NRF1 and KAT2A in the brain tissues of MACO rats and OGD/R-induced RN-c cells, which was elevated by CAT treatment (Fig. 5B–E). As for the results of dual-luciferase reporter gene assay, the luciferase activity of wt-KAT2A promoter co-transfected with oe-NRF1vector was significantly higher than that of wt-KAT2A promoter co-transfected with oe-NC vector, with insignificant difference observed in that of mut-KAT2A (Fig. 5F).

Following sh-NRF1 transfection in normal neurons, western blotting indicated that the sh-NRF1 group had restrained expression of NRF1, KAT2A, and METTL3 and augmented Beclin-1 expression as compared to the sh-NC group (Fig. 5G). With respect to ChIP results, the enrichment level of KAT2A promoter region pulled down by NRF1 antibody was observably decreased after NRF1 knockdown (Fig. 5H), as well as the reduced H3K27AC enrichment level in the METTL3 promoter region (Fig. 5I). Overall, NRF1 activated METTL3 transcription via KAT2A, which may play a protective role in cerebral ischemia.

### CAT relieves OGD/R-induced neuronal injury and excessive autophagy via the NRF1/KAT2A/METTL3 axis

**Fig. 6:**
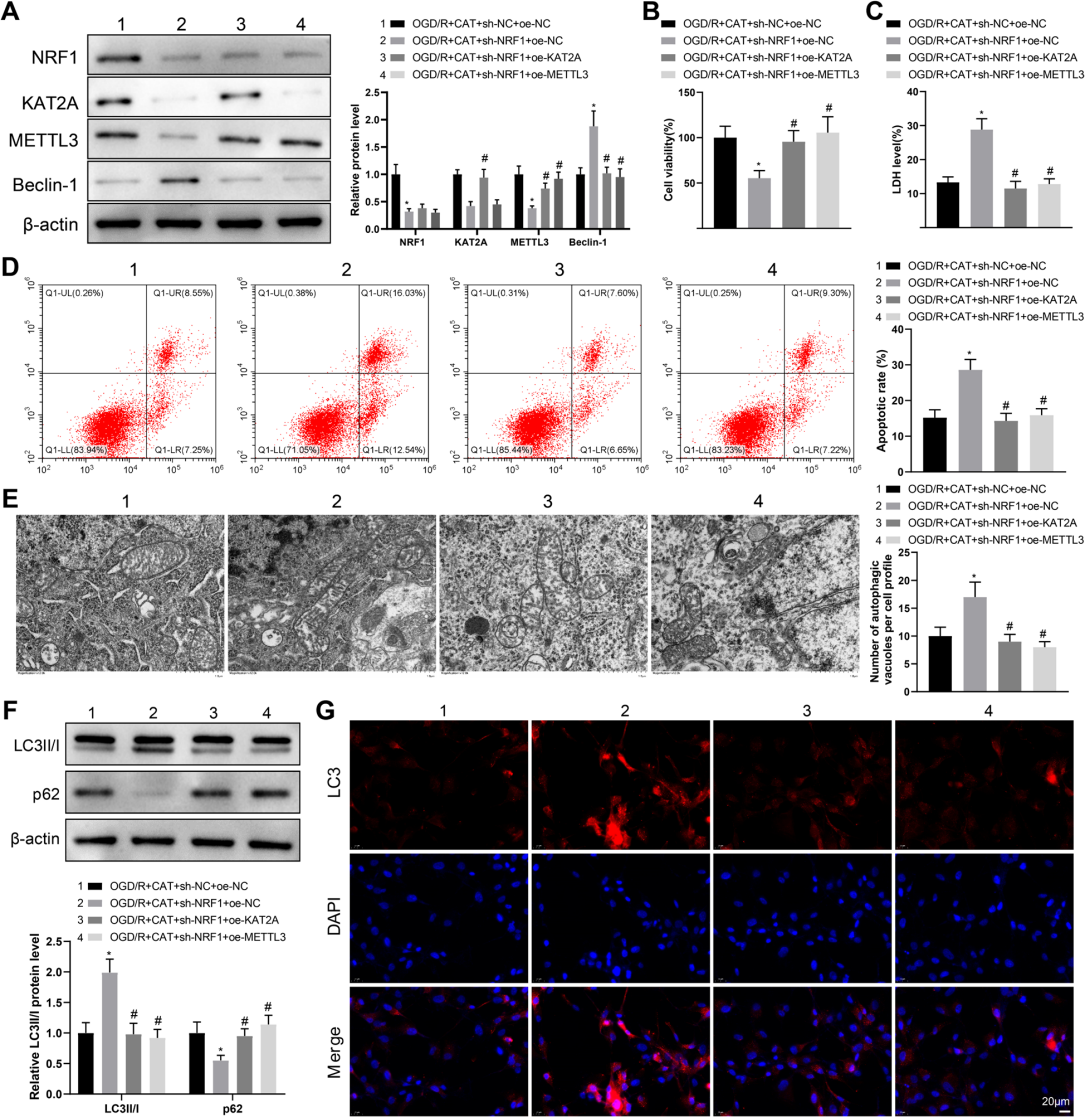
CAT improves OGD/R-induced neuronal injury and excessive autophagy via the NRF1/KAT2A/METTL3 axis. **A** the protein expression of NRF1, KAT2A, METTL3, and Beclin-1 in RN-c cells was measured by western blotting. **B** RN-c cell viability was measured by CCK-8 assay. **C** LDH release was evaluated using the corresponding kit. **D** RN-c cell apoptosis was evaluated by flow cytometry. **E** The number of autophagosomes in RN-c cells was observed through TEM. **F** the expression of LC3II/I and p62 was evaluated by western blotting. **G** the representative images of immunofluorescent staining of LC3. Cell experiments were repeated three times. *P < 0.05, compared with the OGD/R + CAT + sh-NC + oe-NC group; #*P* < 0.05, compared with the OGD/R + CAT + sh-NRF1 + oe-NC group

With intention to elucidate whether CAT modulates cerebral ischemic progression through the NRF1/KAT2A/METTL3/Beclin-1 axis, RN-c cells underwent transfection with sh-NRF1 and oe-KAT2A or sh-NRF1 and oe-METTL3, followed by OGD/R and CAT treatments. In comparison with the OGD/R + CAT + sh-NC + oe-NC group, the expression of NRF1, KAT2A, and METTL3 was substantially reduced but the expression of Beclin-1 was elevated in the OGD/R + CAT + sh-NRF1 + oe-NC group. Relative to the OGD/R + CAT + sh-NRF1 + oe-NC group, the OGD/R + CAT + sh-NRF1 + oe-KAT2A group had increased KAT2A and METTL3 and decreased Beclin-1, while the OGD/R + CAT + sh-NRF1 + oe-METTL3 group had increased METTL3 and decreased Beclin-1 (Fig. 6A).

As displayed in Fig. 6B–D, weakened cell viability and enhanced LDH release and cell apoptosis were found in the OGD/R + CAT + sh-NRF1 + oe-NC group versus the OGD/R + CAT + sh-NC + oe-NC group; however, the OGD/R + CAT + sh-NRF1 + oe-KAT2A and OGD/R + CAT + sh-NRF1 + oe-METTL3 groups exhibited enhanced cell viability and reduced LDH release and cell apoptosis compared with the OGD/R + CAT + sh-NRF1 + oe-NC group. In addition, the OGD/R + CAT + sh-NRF1 + oe-NC group had more autophagosomes than the OGD/R + CAT + sh-NC + oe-NC group, while the OGD/R + CAT + sh-NRF1 + oe-KAT2A and OGD/R + CAT + sh-NRF1 + oe-METTL3 groups had less autophagosomes than the OGD/R + CAT + sh-NRF1 + oe-NC group (Fig. 6E). Also, higher LC3II/I expression but lower p62 expression were discovered in the OGD/R + CAT + sh-NRF1 + oe-NC group than those in the OGD/R + CAT + sh-NC + oe-NC group, whereas the OGD/R + CAT + sh-NRF1 + oe-KAT2A and OGD/R + CAT + sh-NRF1 + oe-METTL3 groups had reverse results versus the OGD/R + CAT + sh-NRF1 + oe-NC group (Fig. 6F). LC3 immunofluorescent staining results further confirmed the autophagy changes (Fig. 6G). Altogether, the above results implicated that CAT palliated OGD/R-induced neuronal injury and excessive autophagy via the NRF1/KAT2A/METTL3 axis.

### CAT alleviates neuronal injury and excessive autophagy in MCAO rats via the NRF1/KAT2A/METTL3/Beclin-1 axis

**Fig. 7:**
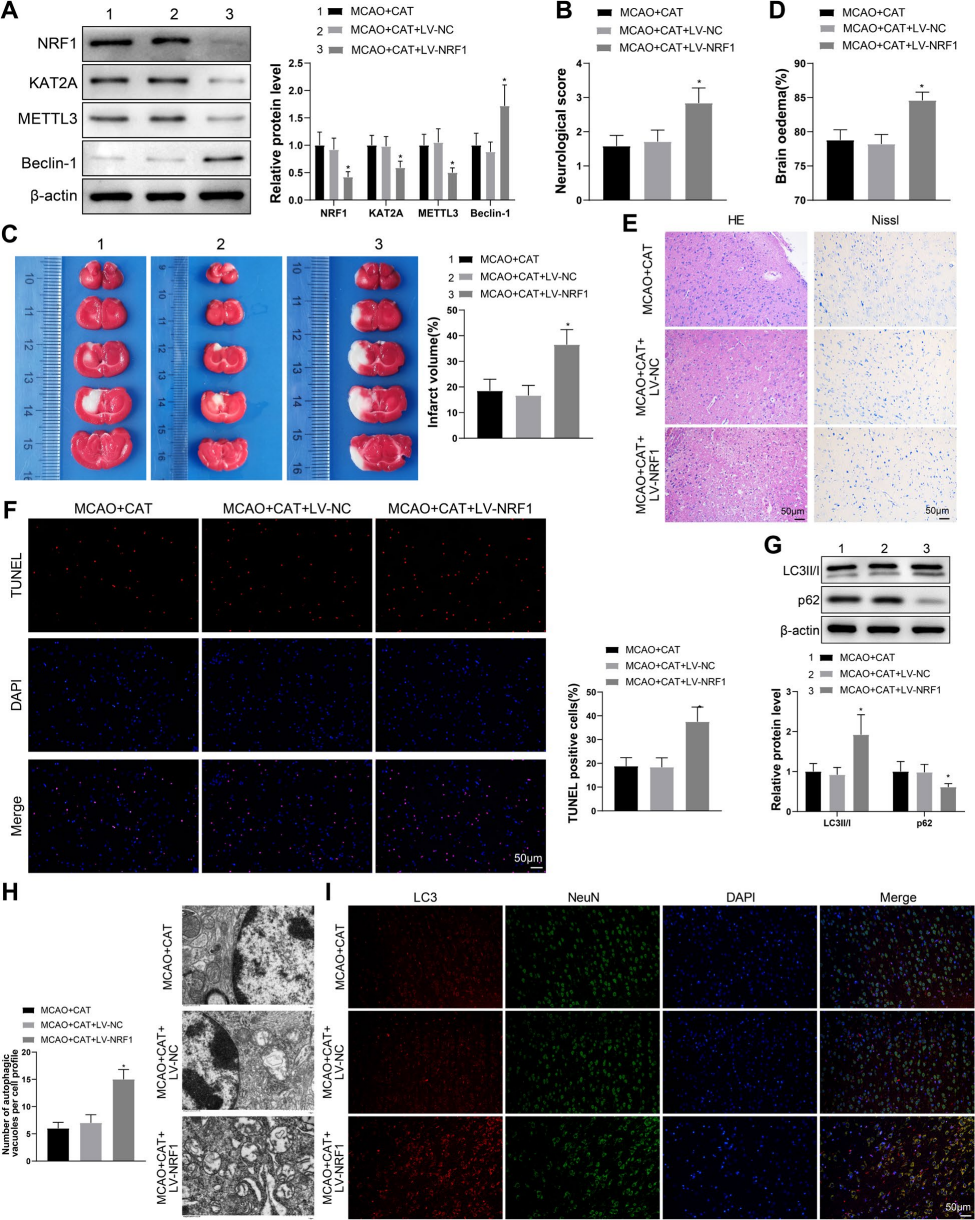
CAT protects against neuronal injury and excessive autophagy in MCAO rats via the NRF1/KAT2A/METTL3/Beclin-1 axis. **A** The expression of NRF1, KAT2A, METTL3, and Beclin-1 was detected by western blotting (n = 6). **B** Neurological deficit score (n = 12). **C** the infarct volume was measured through TTC staining (n = 6). **D** BWC was examined (n = 6). **E** H&E staining and Nissl staining were used to evaluate the morphological changes in the prefrontal cortex (n = 6). **F** TUNEL staining was used to determine the apoptosis of cortical neurons (n = 6). **G** the protein expression of LC3II/I and p62 in ischemic cerebral cortex was tested by western blotting (n = 6). **H** the number of autophagosomes in cortical neurons was observed through TEM (n = 6). **I** the representative images of LC3, NeuN, and DAPI (blue) immunofluorescent staining of cortical areas in each group of rats (n = 6). *P < 0.05, compared with the MCAO + CAT + LV-NC group

Finally, we did animal experiments to explore whether CAT mediated neuronal injury protection and autophagy regulation in MCAO rats through the NRF1/KAT2A/METTL3/Beclin-1 axis. Rats were injected with LV-NRF1 for 3 days, followed by MACO and CAT treatments. Compared with the MCAO + CAT + LV-NC group, the MCAO + CAT + LV-NRF1 group had decreased expression of NRF1, KAT2A, and METTL3 and increased Beclin-1 expression, while there was no significant difference between the MCAO + CAT group and the MCAO + CAT + LV-NC group (Fig. 7A).

Analysis of neurological deficit score revealed that rats injected with LV-NRF1 showed worse neurobehavioral outcomes than normal rats (Fig. 7B). Higher infarct volume and BWC were observed in the MCAO + CAT + LV-NRF1 group than those in the MCAO + CAT + LV-NC group (Fig. 7C, D). From H&E staining and Nissl staining, the MCAO + CAT and MCAO + CAT + LV-NC groups had less cell injury, visible nuclei, and lighter degree of neuronal atrophy, while the MCAO + CAT + LV-NRF1 group had disordered cell arrangement, severely atrophic nuclei, and decreased proportion of Nissl-positive cells, accompanied by atrophied neurons and shrunken nuclei (Fig. 7E). As revealed in Fig. 7F, the MCAO + CAT + LV-NRF1 group had more TUNEL-positive cells than the MCAO + CAT + LV-NC group. There was no significant difference in neurological function between the MCAO + CAT + LV-NC group and MCAO + CAT group (Fig. 7B–F).

Western blotting results unveiled that the MCAO + CAT + LV-NRF1 group had higher LC3II/I expression and lower p62 expression than the MCAO + CAT + LV-NC group (Fig. 7G). The results of TEM and immunofluorescent staining of NeuN and LC3 further confirmed that knockdown of NRF1 could reverse the inhibition effect of CAT on autophagy in MCAO rats (Fig. 7H, I).

In summary, CAT mediated neuronal injury protection and autophagy regulation in MCAO rats via the NRF1/KAT2A/METTL3/Beclin-1 axis.

## Discussion

**Fig. 8:**
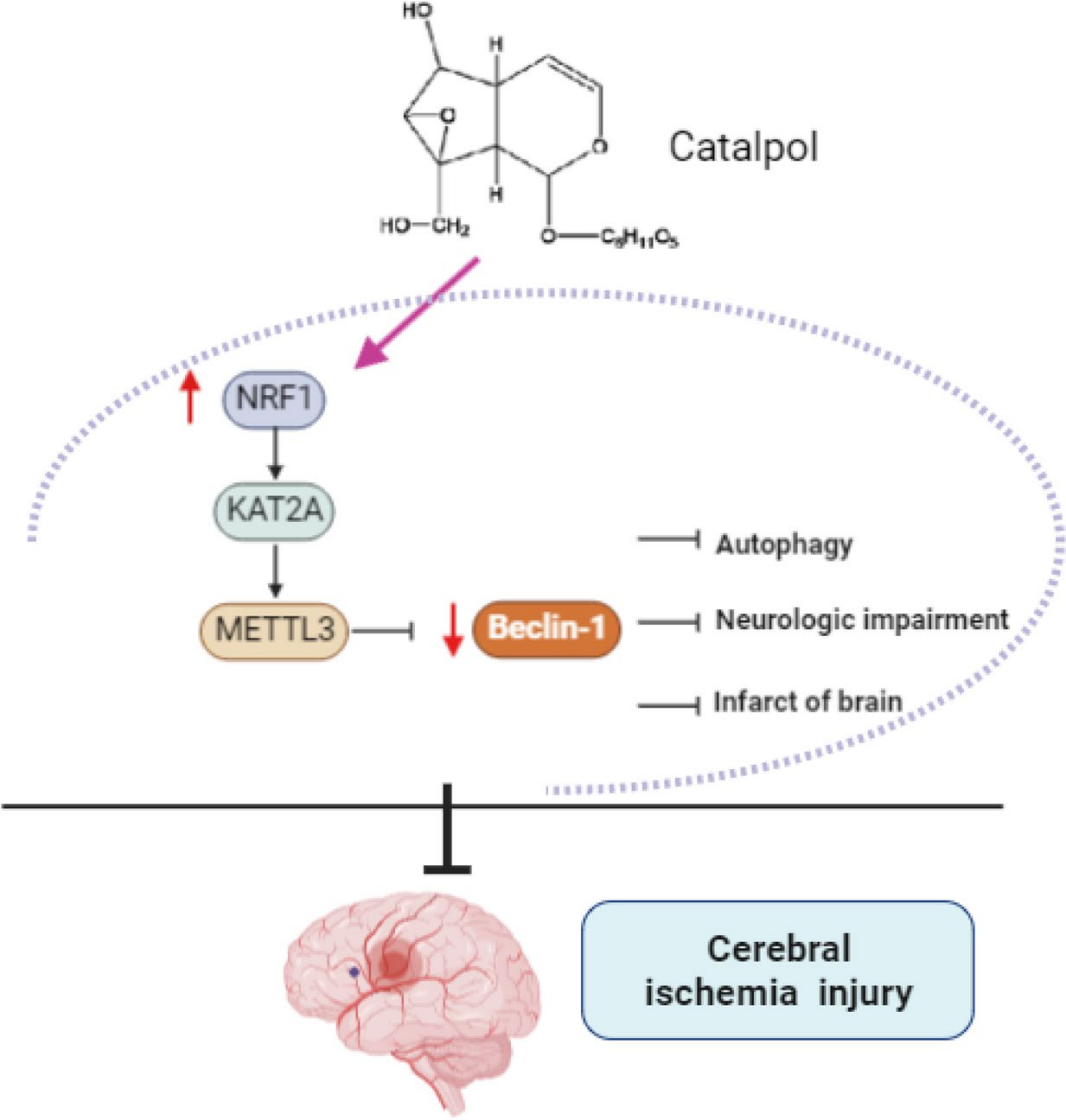
CAT regulates the m^6^A modification of Beclin-1 via the NRF1/KAT2A/METTL3 axis, thereby mediating neuroprotection and autophagy regulation of cortical neurons after cerebral ischemia. CAT treatment could reduce the neurological function deficit and the infarct volume, inhibit neuron cell apoptosis in the cerebral cortex, and significantly improve neuronal injury and excessive autophagy in MCAO rats 7 days after ischemia. Mechanistically, CAT upregulates NRF1, promotes KAT2A transcription, and activates METTL3, and the activated METTL3 inhibits Beclin-1 expression by mediating m^6^A modification of Beclin-1, ultimately exerting the protective effect on cerebral ischemia progression

In recent years, the study on the mechanism of drug combining signaling pathway has become the mainstream in cerebral ischemia. For instance, Yang et al. pointed out that *Arctium lappa L. roots* protected against cerebral ischemia by repressing apoptosis and AMPK/mTOR-mediated autophagy (Yang et al. 2021a, b). Jiang and his team specified that *Artemisinin* could attenuate OGD/R-induced oxidative stress damage through PHB2-mediated mitophagy (Jiang et al. 2022). The active compounds in drugs can regulate the autophagy of various cells, including tumor cells, neurons, myocardial cells, and endothelial cells (Huang et al. 2015). From this perspective, the effective mechanism affecting the autophagy of neurons is sought to provide a theoretical basis for drug research in cerebral ischemia. As a result, the present study revealed that CAT, the main active component of *Rehmannia*, protected against cerebral ischemia in vivo and in vitro by inhibiting neuronal injury and autophagy via the NRF1/KAT2A/METTL3/Beclin-1 axis (Fig. 8).

At first, we established a rat model of MCAO with Sprague–Dawley rats and a cell model of OGD/R in RN-c cells. Increased neurological deficit score, infarct volume, BWC, neuronal apoptosis, autophagosomes, and LC3II/I expression and decreased neuronal viability and p62 expression were observed in MCAO rats or OGD/R-induced RN-c cells, accompanied by elevated Beclin-1 expression. Beclin-1 is regarded as a major regulator of autophagy, which playing a fatal role in human neurodegenerative diseases, including cerebral ischemia (Mo et al. 2020; Xu and Qin 2019). Increasing evidence showed that autophagy can be evaluated by detecting Beclin-1, LC3 and p62 expression (Yang et al. 2021a, b; Yilmaz et al. 2023). According to our results, CAT treatment alleviated neuronal injury, decreased MCAO or OGD/R-induced Beclin-1 expression and LC3II/I ratio, and increased p62 level, while Beclin-1 overexpression nullified the protection of CAT in OGD/R-induced RN-c cells. A previous study documented that melatonin repressed OGD/R challenged SH SY5Y cell autophagy, as evidenced by the reduction of Beclin-1 (Zhi et al. 2020). These results suggested that the protective mechanism of CAT against neuronal injury may be related to autophagy inhibition in cerebral ischemia. A previous review showed that CAT exhibited anti-ischemic activity by promoting neuroprotection and neural repair (Zhu et al. 2022), so it has become the focus of many researchers in the field of cerebral ischemia. For example, Xie et al. found that CAT could improve the ultrastructure morphology of neurons and further protect limb motor function and hippocampal neurological functions in cerebral ischemia rats (Xie et al. 2022). Additionally, a prior study displayed that CAT played a pleiotropic neuroprotective role in cerebral ischemia by inhibiting oxidation, apoptosis, inflammation, and autophagy (Zheng et al. 2017), but its mechanism has not been studied. In our paper, we further verified that CAT treatment alleviated neuronal injury and autophagy in vivo and in vitro by declining the expression of Beclin-1.

To deeply ascertain the upstream genes of Beclin-1, we noted that METTL3 was downregulated in MCAO rats and OGD/R-induced RN-c cells, and METTL3 could negatively Beclin-1 expression via m^6^A modification. A study by Chang et al. emphasized that importance of m^6^A modification in the pathogenesis of cerebral ischemia (Chang et al. 2022). Mechanistically, METTL3 decreased the expression of ANXA2 in T lymphocytes via m^6^A modification to reduce brain injury in MCAO rats and neuronal damage in OGD/R-exposed neurons (Liu et al. 2023). Another study unveiled that METTL3 deficiency could enhance autophagic flux in hypoxia/reoxygenation-treated cardiomyocytes (Song et al. 2019). Consistently, our findings demonstrated that CAT reduced OGD/R-induced neuronal injury and excessive autophagy via METTL13-inhibited Beclin-1. Moreover, bioinformatics data and experimental results implied that NRF1 activated METTL3 transcription via KAT2A, which assumed a neuroprotective role in cerebral ischemia. Specifically, inhibition of NRF1 abrogated the protective effect of CAT on neuronal injury and excessive autophagy, which could be restored by overexpression of KAT2A or METTL3. Acetylation has emerged as an important regulatory mechanism for autophagy (Xu and Wan 2023). KAT2A as an acetyltransferase was verified to be closely linked to autophagy regulation (Ouyang et al. 2020). A previous discovery showed that electromagnetic stimulation activated KAT2A, which increased hippocampal neurogenesis in aged and Hutchinson-Gilford progeria mouse brains, thereby remitting the symptoms of aging (Chang et al. 2021). NRF1 is identified as a protective factor in cerebral ischemia progression (Wang et al. 2023). Upregulated NRF1 is associated with ameliorated depressive-like behaviors and improved cognitive dysfunctions in electroacupuncture-treated mice with post-stroke depression (Xia et al. 2017). Molecularly, the main regulator of mitochondrial biogenesis PGC-1α improved mitochondrial function by activating its downstream genes NRF1 and TFAM, thereby remitting cerebral ischemia–reperfusion injury (Yuan et al. 2023). More importantly, compelling evidence elicited that CAT enhanced mitochondrial biogenesis, evidenced by elevations in the expression of PGC-1α, NRF1, and TFAM (Xu et al. 2020). Finally, we conducted in vivo and in vitro assays and confirmed that CAT remitted neuronal injury and excessive autophagy via the NRF1/KAT2A/METTL3/Beclin-1 axis.

## Conclusion

Collectively, our findings indicated that the neuroprotective effect of CAT against cerebral ischemia was associated with the inhibition of autophagy via the NRF1/KAT2A/METTL3/Beclin-1 axis. Certainly, this work still has some limitations. It is important to note that our study used 7-week-old rats, and cerebral ischemia occurs more often in older people. Therefore, the therapeutic effect of CAT on cerebral ischemia in elderly rodents needs further experimental research. In addition, glial cells and endothelial cells are also important components of brain tissue, and whether the protective effect of CAT on glial cells and endothelial cells is similar to the protective effect on neurons needs to be further verified. Of course, CAT cannot be used as a specific drug, but it can be an indispensable adjunct to the treatment of cerebral ischemia. Therefore, the therapeutic mechanism of CAT in stroke still needs to be further improved to provide a solid foundation for its future clinical application.

## Data Availability

The datasets used or analyzed during the current study are available from the corresponding author on reasonable request.
